# GOIZ ZAINDU study: a FINGER-like multidomain lifestyle intervention feasibility randomized trial to prevent dementia in Southern Europe

**DOI:** 10.1186/s13195-024-01393-z

**Published:** 2024-02-27

**Authors:** Mikel Tainta, Mirian Ecay-Torres, Maria de Arriba, Myriam Barandiaran, Ane Otaegui-Arrazola, Ane Iriondo, Maite Garcia-Sebastian, Ainara Estanga, Jon Saldias, Montserrat Clerigue, Alazne Gabilondo, Naia Ros, Justo Mugica, Aitziber Barandiaran, Francesca Mangialasche, Miia Kivipelto, Arantzazu Arrospide, Javier Mar, Pablo Martinez-Lage, on behalf of the GOIZ ZAINDU study group, I. Aquizu, M. A. Arrondo, E. Baztarrika, L. Etxeberria, E. García-Arrea, M. García-Domínguez, E. Imaz, M. Iparragirre, M. Iridoy, A. Larrea, M. D. López, F. Martin, A. Olaskoaga, P. Pacheco, A. M. Pérez-Rodiguez, Y. Porres, M. Ruibal, B. San Juan, M. J. Tilves, E. Zapirain

**Affiliations:** 1 CITA-alzheimer Foundation, Donostia-San Sebastián, País Vasco, Spain; 2 Osakidetza, Organización Sanitaria Integrada (OSI) Goierri-Urola Garaia, País Vasco, Spain; 3 Osakidetza, Organización Sanitaria Integrada (OSI) Donostialdea, País Vasco, Spain; 4 Osakidetza, Organización Sanitaria Integrada (OSI) Bidasoa, País Vasco, Spain; 5 Osakidetza, Organización Sanitaria Integrada (OSI) Debagoiena, País Vasco, Spain; 6 Instituto de Investigación Sanitaria Biodonostia, Donostia-San Sebastián, Spain; 7 Instituto de Investigación en Servicios Sanitarios Kronikgune, Barakaldo, Spain; 8 University of the Basque Country UPV/EHU, Donostia-San Sebastian, Spain; 9 Division of Clinical Geriatrics, Center for Alzheimer Research, Department of Neurobiology, Care Sciences and Society, Karolinska Institutet, Stockholm, Sweden; 10 Medical Unit Aging, Theme Inflammation and Aging, Karolinska University Hospital, Stockholm, Sweden; 11 The Ageing Epidemiology Research Unit, School of Public Health, Imperial College London, London, UK; 12 Institute of Public Health and Clinical Nutrition, University of Eastern Finland, Kuopio, Finland

**Keywords:** Cognitive impairment, Dementia prevention, Lifestyle, Multidomain intervention, Randomized trial

## Abstract

**Background:**

GOIZ ZAINDU (“caring early” in Basque) is a pilot study to adapt the Finnish Geriatric Intervention Study to Prevent Cognitive Impairment and Disability (FINGER) methodology to the Basque population and evaluate the feasibility and adherence to a FINGER-like multidomain intervention program. Additional aims included the assessment of efficacy on cognition and data collection to design a large efficacy trial.

**Method:**

GOIZ ZAINDU is a 1-year, randomized, controlled trial of a multidomain intervention in persons aged 60+ years, with Cardiovascular Risk Factors, Aging and Dementia (CAIDE) risk score ≥ 6, no diagnosis of dementia, and below-than-expected performance in at least one of three cognitive screening tests. Randomization to a multidomain intervention (MD-Int) or regular health advice (RHA) was stratified by sex, age (>/≤ 75), and cognitive status (mild cognitive impairment (MCI)/normal cognition). MD-Int included cardiovascular risk factor control, nutritional counseling, physical activity, and cognitive training. The primary outcomes were retention rate and adherence to the intervention program. Exploratory cognitive outcomes included changes in the Neuropsychological Test Battery *z*-scores. Analyses were performed according to the intention to treat.

**Results:**

One hundred twenty-five participants were recruited (mean age: 75.64 (± 6.46); 58% women). The MD-Int (*n* = 61) and RHA (*n* = 64) groups were balanced in terms of their demographics and cognition. Fifty-two (85%) participants from the RHA group and 56 (88%) from the MD-Int group completed the study. More than 70% of the participants had high overall adherence to the intervention activities. The risk of cognitive decline was higher in the RHA group than in the MD-Int group in terms of executive function (*p* =.019) and processing speed scores (*p* =.026).

**Conclusions:**

The GOIZ-ZAINDU study proved that the FINGER methodology is adaptable and feasible in a different socio-cultural environment. The exploratory efficacy results showed a lower risk of decline in executive function and processing speed in the intervention group. These results support the design of a large-scale efficacy trial.

**Trial registration:**

GOIZ ZAINDU feasibility trial was approved and registered by the Euskadi Drug Research Ethics Committee (ID: PI2017134) on 23 January 2018. Retrospectively registered in ClinicalTrials.gov (NCT06163716) on 8 December 2023.

**Supplementary Information:**

The online version contains supplementary material available at 10.1186/s13195-024-01393-z.

## Background

The increase in life expectancy of the population is one of the most remarkable counterparts in medicine and social progress. However, since aging is the main risk factor for dementia, an increase in the number of older adults is linked to an increase in the number of people living with disabilities, including dementia. According to the last Global Burden of Disease Study [1], the number of people living with dementia has more than doubled from 1990 to 2016. The World Health Organization (WHO) in the “Global status report on the public health response to dementia” has estimated that people living with dementia could be about 139 million by 2050. Forecasting models for the future burden of dementia in many countries predict an unmanageable growth in the number of cases if effective prevention initiatives are not developed [2].

Dementia is a multifactorial process influenced by genetic and environmental conditions and results from lifelong interactions between protective and risk factors [3]. Midlife modifiable dementia risk factors such as cardiovascular health, physical inactivity, depression, and low education may account for up to a third of the cases of dementia worldwide [4]. As different degrees of exposure to these factors can modify the plastic trajectories of aging [3, 5], a window of opportunity for research on prevention is open [5, 6]. Achievements in the control and promotion of cardiovascular health along with improvements in population education levels are probably behind the apparent reduction of dementia occurrence in some developed countries [2, 7, 8].

Different scores and indexes have been proposed to estimate individual dementia risk based on risk factors. The Cardiovascular Risk Factors, Aging and Dementia (CAIDE) risk score [9] is a well-known validated tool able to predict cognitive trajectories, neurodegeneration, and amyloid deposition [10, 11]. The CAIDE score has also been used to identify and enroll in prevention initiatives for at-risk individuals with modifiable conditions [12]. The Finnish Geriatric Intervention Study to Prevent Cognitive Impairment and Disability (FINGER, ClinicalTrials.gov Identifier: NCT01041989) [12] is the first randomized clinical trial showing that a multidomain, lifestyle-based intervention benefits cognition in persons with increased CAIDE risk score. Despite their primary negative results, other multidomain European trials (the French Multidomain Alzheimer Preventive Trial - MAPT, and the Dutch Prevention of Dementia by Intensive Vascular care - PreDIVA) have confirmed that interventions on risk and protective factors represent a window of opportunity for dementia prevention in participants with increased risk and frailty [13–15].

To fully understand the impact of such preventive interventions, their feasibility and efficacy must be tested worldwide. To this aim, the World-Wide FINGERS network of multidomain trials for dementia risk reduction and prevention was established to explore the feasibility and efficacy of multidomain lifestyle interventions [16] in different populations, regions, and social contexts worldwide. Previous experiences [17] have shown the importance of conducting a pilot study to adapt the “FINGER-like” methodology and obtain data on which to base the design of a large-scale efficacy study. Here, we present the GOIZ ZAINDU (“caring early” in Basque) multidomain intervention pilot study results. GOIZ ZAINDU is a feasibility study to adapt the FINGER trial methodology to a southern European context, in a real clinical practice setting. We evaluated the applicability and adherence to a FINGER-like intervention and explored the effect of the multidomain intervention on cognitive performance in older adults after one year.

## Methods

### Study design and participants

The GOIZ ZAINDU pilot trial is a feasibility study of a 1-year controlled, randomized, multidomain intervention trial, for prevention of cognitive decline, carried out in the municipality of Beasain in the Basque Country (Spain). Participants were recruited in collaboration with the primary care center health providers and the Municipality of Beasain after an informative lifestyle and dementia prevention campaign.

Participants were at least 60 years of age and had a CAIDE score ≥ 6 points. Additionally, they scored below the cut-off points for our population [18] in at least one of two brief cognitive tests—Memory Alteration Test, “T@M” [19], and Fototest [20]—or had a score of 2 or higher in the AD8 informant’s questionnaire [21] of cognitive symptoms.

The exclusion criteria included the presence of uncontrolled cardiovascular or respiratory disease, previous diagnosis of dementia, ongoing neurological disorders, unstable psychiatric disease, evidence of any other severe disease of any etiology, or any situation in the investigator’s opinion that could compromise safe engagement in the intervention.

GOIZ ZAINDU feasibility trial was approved by the Euskadi Drug Research Ethics Committee (ID: PI2017134). All the participants provided written informed consent at the screening visit. This pilot randomized trial was conducted following the Consolidated Standards of Reporting Trials guidelines (CONSORT) [22] and the CONSORT extensions for pilot abstract and pilot trials [23].

### Screening evaluation

The participants’ demographic information included age, sex, race/ethnicity, native language, and years of education. During the screening visits, assessments were conducted to ensure the fulfillment of the inclusion criteria and the absence of the exclusion criteria. The participants’ general practitioners (GP) collected data on medical conditions and medications. The CAIDE dementia risk score was calculated, and the T@M, Fototest, and AD8 questionnaires were administered by trained psychologists in the municipality of Beasain.

### Baseline visit and diagnostic workout evaluation

Pre-selected participants in the screening phase underwent a clinical evaluation and a physical, cognitive, and behavioral assessment to ensure the completion of the study assessment. Dementia cases, defined by the DSM-IV, were excluded from the study, and the diagnosis of mild cognitive impairment (MCI) [24] cases were ascertained.

### Randomization and masking

After the baseline evaluation, all participants received verbal information regarding the potential benefits of caring for vascular risk factors, adherence to the Mediterranean diet, and good cognitive and physical activity routines. Participants were randomly assigned to either a standard health advice control group (RHA, control) or a multidomain intervention group (MD-Int). Random assignment followed a proportion of 1:1 and was stratified by age (< 75 vs. ≥ 75 years), sex, and cognitive status (normal cognition vs. MCI). Randomization was independently carried out by researchers from the Research Unit of the Basque Health System using a computerized application based on obtaining random numbers. Double blinding is challenging to achieve in this type of studies. Nevertheless, the participants were not informed of the specific group to which they were assigned. They were urged not to comment on program details during the evaluation sessions.

## Intervention period (summarized in Fig. 1)

**Fig. 1:**
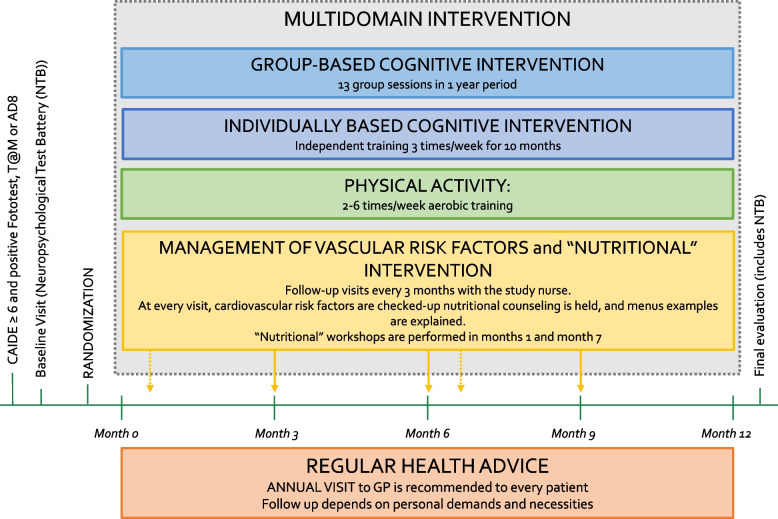
GOIZ ZAINDU study design

1. Regular health advice control (RHA) group. Participants randomized to the control group followed preventive programs already ongoing in their primary care center. These included individual visits to reinforce tabaco quitting and annual group sessions to underline the importance of physical activity, socialization, smoking, healthy diet, and alcohol usage. Visits to the GP and nurse depended on personal demands and necessities. The general recommendation from the Basque Public Health System is to receive an annual consultation with the GP for all patients over 60 years old.
2. Multidomain intervention (MD-Int) group. The MD-Int program was designed to provide tools and routines that participants could incorporate into their daily living activities. Close relatives of the participant were encouraged to get involved in the activities to be carried out at home, such as preparing the weekly menu and carrying out individual cognitive training tasks. Participation in group activities designed for the intervention, with people from the same municipality was prompted to reinforce and enrich the social environment of the participants. Although the program included standardized guidelines and exercises, each participant was considered individually, adapting nutritional requirements and physical and cognitive activities according to individual needs and abilities. This methodology is based on the FINGER trial design [23] but has been adapted to local resources and the healthcare system. GPs and nurses were involved in follow-up visits. Most intervention activities were conducted at the local primary care center. Local town hall resources such as group activities for older adults at the municipality sports center and current outdoor sports activities were incorporated in the study. The MD-Int program included (1) individual follow-up visits every 3 months for cardiovascular risk factor monitoring and nutritional counseling with primary health care providers, (2) two nutritional workshops led by a nutritionist, (3) 20 h of cognitive stimulation delivered through group sessions, and (4) 40 h of individual cognitive training exercises. Participants in the MD-Int group received recommendations to practice 2–6 h of physical exercise per week and were involved in sports activities. Social stimulation was promoted through group activities.
  A. Intensive control and monitoring of cardiovascular risk factors: Every 3 months, a follow-up visit was performed individually at the primary care unit for cardiovascular risk factor check-up, including measurements of blood pressure, pulse, height, weight, hip, and waist circumference. During these visits, participants were reminded of the study objectives and motivated to adhere to them. Whenever a poorly controlled or newly detected risk factor was detected, advice and recommendations for adequate control, initiation, or adjustment of pharmacological treatments were provided by the participants’ GP.
  B. Nutritional counseling was based on the Mediterranean Diet pattern [25, 26]. Two workshops were conducted by the nutritionist at the beginning and in the middle of the intervention period. Individual sessions with verbal and written counseling were administered during follow-up visits. The materials given to participants included example menus to increase adherence to the Mediterranean diet. At baseline and 12-month visits and every 3 months, a 14-item dietary questionnaire [27] to assess adherence to the Mediterranean diet was recorded.
  C. Physical activity and exercise. Participants were encouraged to remain physically active during the baseline and every follow-up visit. The recommendations were based on the American Heart Association Guidelines and the ICOPE (Integrated Care for Older People) Guidelines from the WHO. Participants were encouraged to sign up for the city council’s outdoor aerobic physical activity programs such as hiking or Nordic walking. Indoor group activities were organized and guided by personnel from the Municipal Sports Center twice a week during an intervention period of 9 months.
  D. Cognitive interventions were divided into individual sessions and 13 group activities. The main goal was to incorporate cognitively stimulating daily habits and routines and emphasize the family and social environment. Group sessions lasted 90 min. and were guided by a neuropsychologist and included several topics, such as age-related cognitive changes, learning strategies for activities of daily living, and knowledge of self-cognition. For 10 months of the intervention period, individual work was designed to be completed by subjects within 20 min, three times per week. This paper material was based on the NeuronUP© platform and was specially designed for non-demented people and adapted to every participant according to three characteristics: cognitive status, education, and current or past (if retired) occupational level based on Hollingshead Index for socioeconomical status. The objective was to train and reinforce executive function, visuospatial skills, language, episodic memory, and working memory. This material was completed using EXERCITA© cognitive training materials that were specifically developed considering the cultural and linguistic context of the Basque Country population Fig. 1.

### Feasibility outcomes

The primary objective of this study was feasibility. Therefore, adherence to the intervention and retention rates were the primary outcomes. Retention rate was defined as the proportion of participants who completed the 12-month trial period. Regarding dropouts, we considered a discontinuation rate of less than 20% satisfactory. Trial sample size was similar to previous multidomain feasibility randomized trials [17].

Adherence to each intervention component was based on participation in the activities offered in the intervention group. Study coordinators assessed adherence to intervention activities by recording the number of workshops and follow-up visits attended and by checking the cognitive training workbook. Self-reported information on weekly physical activity and attendance to group activities at the sports center was recorded for physical exercise.

To evaluate overall adherence to intervention activities, we used a semi-quantitative scale. Table 1 shows the scoring of each intervention component. We simultaneously considered both the degree of adherence to each intervention component and the degree of attendance for each intervention. Overall, “high adherence” was considered when the attendance of all intervention components was higher than 50%. “Partial adherence” was defined by attendance to at least 30% of activities of all intervention components. Overall, “low adherence” was considered when attendance to any intervention components was lower than 30%. Attendance of less than 30% to two or more intervention components was considered “very low adherence” overall adherence.

**Table 1:** Semi-quantitative scale to estimate overall adherence

**Overall adherence**	**Cardiovascular follow-up visits attendance**	**Nutrition visits attendance**	**Cognitive intervention workshops attendance**	**Cog.training individual materials completed**	**Physical exercise completed**	**No. participants (%)**
**High adherence** (all criteria must be met)	At least 2 of 3	At least 2 of 3	≥ 50%	≥ 50%	≥ Twice a week	35 (54.71%)
**Partial adherence** (all criteria must be met)	At least 1 of 3	At least 1 of 3	≥ 30%	≥ 30	≥ Twice a week	11 (17.18%)
**Low adherence**	Less than 30% of attendance/completion in ANY intervention domain					12 (18.75%)
**Very low adherence**	< 30% of attendance/completion in ALL intervention domains					6 (9.36%)

Adherence rates were calculated for the entire follow-up period, including external factors of the intervention program, such as medical and family issues and COVID-19 outbreak social distancing measures, to obtain a realistic picture of the potential implementation and maintenance of this type of intervention in a real scenario. Demographics and cognitive status at baseline were analyzed as predictors of participant adherence.

### Efficacy exploratory outcomes

Cognitive performance was assessed at baseline and 12 months using the modified Neuropsychological Test Battery (NTBm) [28, 29], which includes the following tests: Wechsler Memory Scale-III Logical Memory, Consortium to Establish a Registry for Alzheimer’s Disease (CERAD) Word List, WMS-R Visual Paired Associates, Category Fluency, Wechsler Adult Intelligence Scale-III Digit Span, Concept Shifting Test, Trail Making Test, shortened 40-stimuli version of Stroop Test, and Letter Digit Substitution Test.

### Additional information

At baseline and final evaluations, additional information was collected on global cognition measured with the Mini-Mental State Examination (MMSE) [30], occupation level was classified according to the Hollingshead Four Factor Index of Social Status, depression and anxiety symptoms were assessed using the Hospital Anxiety and Depression Scale (HADS) [31], and physical fitness was measured using the 6 min walking test [32].

### Safety assessments

Information was obtained and confirmed from the participant GPs regarding current diagnoses, medications, and laboratory values (blood count, cholesterol, glucose, renal and liver function, thyroid hormones, B_12_ vitamin, folic acid) before the start of the intervention period. A structured interview for adverse events was conducted at every follow-up visit.

### Statistical analysis

Variables were checked for normal distribution. Independent samples *t*-test, Mann-Whitney test, and *χ*^2^, as appropriate, were conducted to compare demographics, psychological symptoms, and cognitive performance between the MD-Int and RHA groups at pre-intervention and post-intervention visits.

NTBm *z*-scores were calculated [12] and standardized to the baseline mean and SD, with higher scores suggesting better performance. Five cognitive domain indexes were created: NTBm total score based on the 14 tests (Table 2S), executive functioning domain based on five tests, processing speed domain based on three tests, and memory domain based on six tests (memory global). The minimum number of necessary NTB components was set to eight of 14 for calculating the NTB total score, three of five for executive functioning, two of three for processing speed, and three of six for memory.

As an exploratory objective, the mean change in *z*-scores between pre- and post-intervention visits was calculated for each group and compared between both groups using an independent samples *t*-test. Mixed models of repeated measures were conducted considering the two evaluations made on the study participants to assess the intervention effect on the *z*-scores. Binary logistic analyses were carried out to analyze the risk of cognitive decline in the Standard Health Advice control group compared with the multidomain intervention group. Cognitive decline was defined as a decrease in NTBm scores between pre- and post-intervention assessments. Mixed models and logistic binary regressions were repeated, introducing the level of education and any variables showing significant differences in group comparisons as covariables. Analyses were performed according to the intention to treat. All statistical analyses were performed using SPSS version 20 (SPSS Inc., Chicago, IL, USA). Mixed models were created using STATA.

## Results

**Fig. 2:**
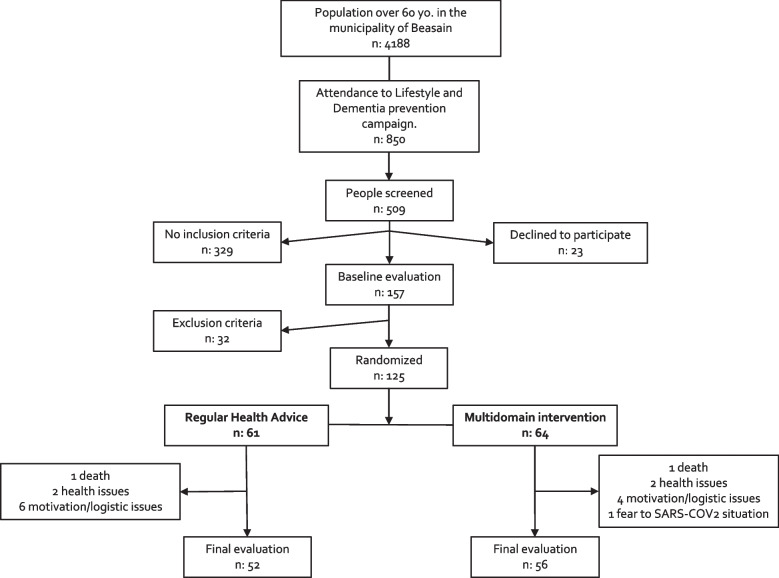
GOIZ ZAINDU study participant CONSORT flowchart

In early 2017, Beasain municipality had over 4100 people aged 60+ years. The GOIZ-ZAINDU study recruitment period began in March 2017. In total, 509 individuals were screened in March 2018. A total of 180 patients fulfilled the inclusion criteria, 23 declined to participate, and 32 met at least one exclusion criterion, mostly dementia. The intervention period lasted from October 2018 to November 2019. One hundred twenty-five subjects were randomized to the MD-Int. group (*n*: 64) or the RHA group (*n*: 61). (Fig. 2). Of these, 108 (86%) participants completed the post-intervention assessment (the retention rate by group was 88% in the MD-Int arm and 85% in the RHA arm). Due to the COVID outbreak and lockdown period in Spain (which started in March 2020), 14 post-evaluation assessments were delayed by 5 months from February to July 2020, five from the RHA group, and nine in the MD-Int group. As shown in Fig. 2, dropout rates were similar in both groups. The main reasons were lack of time or difficulties in participation (10 participants), health-related reasons (4 subjects), and one subject declined to perform post-intervention evaluation because of fear of COVID-19. Two individuals, one in each group, died during the study. No adverse events related to the study procedure were observed.

The mean age was 75.64 (SD 6.46), range 60 to 90 years. Years of education and the distribution of employment levels were expected for the population of this generation in an industrial town in our country. Both groups were balanced in terms of demographics, distribution of dementia risk factors, cognitive performance, and the presence of MCI (Table 2 and Table 2S). The adherence scores to the Mediterranean Diet and physical fitness (distance walked in 6 min) were slightly higher in the MD-Int group (*p* < 0.05).

**Table 2:** Pre-intervention characteristics per group

**Characteristics at baseline**	**Regular health advice (*n* = 61)**	**Multidomain intervention (*n* = 64)**	*p*
**Demographic characteristics**			
Age	76.07 (6.68)	75.22 (6.26)	0.469
Age range	60.67–90.58	59.50–88.33	
Sex: woman, *n* (%)	36 (59.01%)	37 (57.81%)	0.518
Education (years)	7.72 (2.92)	8.48 (4.11)	0.305
Occupation			
Upper	2 (3)	10 (16)	0.060
Middle	24 (39)	22 (35)	
Lower	35 (57)	31 (49)	
**CAIDE dementia risk score**	9 (7–10)	9 (7–11)	0.540
**Vascular factors**			
Systolic blood pressure (mmHg)	142.09 (21.30)	135.92 (15.70)	0.075
Diastolic blood pressure (mmHg)	76.64 (14.78)	76.62 (8.92)	0.275
Serum total cholesterol (mmol/l)	4.83 (1.03)	5.11 (1.19)	0.171
Fasting plasma glucose (mmol/l)	5.87 (0.93)	5.71 (0.60)	0.506
Body mass index (kg/m^2^)	27.28 (3.69)	28.30 (4.51)	0.268
Waist perimeter (cm)	96.36 (7.97)	97.69 (12.48)	0.491
Hip perimeter (cm)	103.26 (7.82)	104.54 (9.38)	0.423
Physical performance (6 min walking test)	480 [386.50–514]	495 [440–542]	0.045
**Lifestyle-related factors**			
Current and former smokers (%)	24 (39.34%)	25 (39.06%)	0.651
Mediterranean diet adherence score (PREDIMED)	7 [6–8]	8 [7, 8]	0.040
Leisure activities questionnaire [33]	27.97 (6.35)	28.48 (6.34)	0.656
Productive activities questionnaire^a^	15.48 (5.97)	16.25 (4.82)	0.435
**Medical conditions**			
Hypertension (%)	34 (55.73%)	32 (50%)	0.521
Hypercholesterolemia (%)	28 (45.59%)	31 (48.44%)	0.713
Diabetes (%)	15 (24.59%)	12 (18.75%)	0.428
History of myocardial infarction (%)	5 (8.20%)	3 (4.69%)	0.457
History of stroke (%)	4 (6.56%)	3 (4.69%)	0.649
Anxiety (HADS)	4 [2–8]	6 [3–8]	0.107
Depression (HADS)	2 [1–5]	3 [1–6]	0.175
**Cognition**			
Mild cognitive impairment (%)	19 (31.15%)	25 (39.06%)	0.354
Mini Mental State Examination	27 [25–29]	26 [24–28]	0.709
NTB global *z* score	0.02 (0.67)	− 0.07 (0.72)	0.482
NTB memory *z* score	0.04 (0.74)	− 0.04 (0.84)	0.555
NTB executive functioning *z* score	0.04 (0.67)	− 0.10 (0.72)	0.258
NTB processing speed *z* score	0.03 (0.84)	− 0.05 (0.92)	0.608

Mean (SD) and median [IQR] of measures unless categorical variables are given in number (%). Independent-samples *t*-tests, Mann-Whitney test, and *χ*^2^ tests were conducted. *HADS* Hospital Anxiety and Depression Scale; *NTBm* Modified Neuropsychological Test Battery

^a^Based on Hollingshead Index for socioeconomical status

The adherence rates to the individual components of the intervention are presented in Table 1S. 67.2% and 73.4% of the subjects completed at least 2/3 of the cardiovascular monitoring and nutritional counseling visits, respectively. 64.1% of the participants completed more than 50% of the cognitive training individual materials, and 70% attended more than half of the cognition workshops. Over 75% of the participants reported practicing physical exercise at least twice a week during the intervention period.

**Fig. 3:**
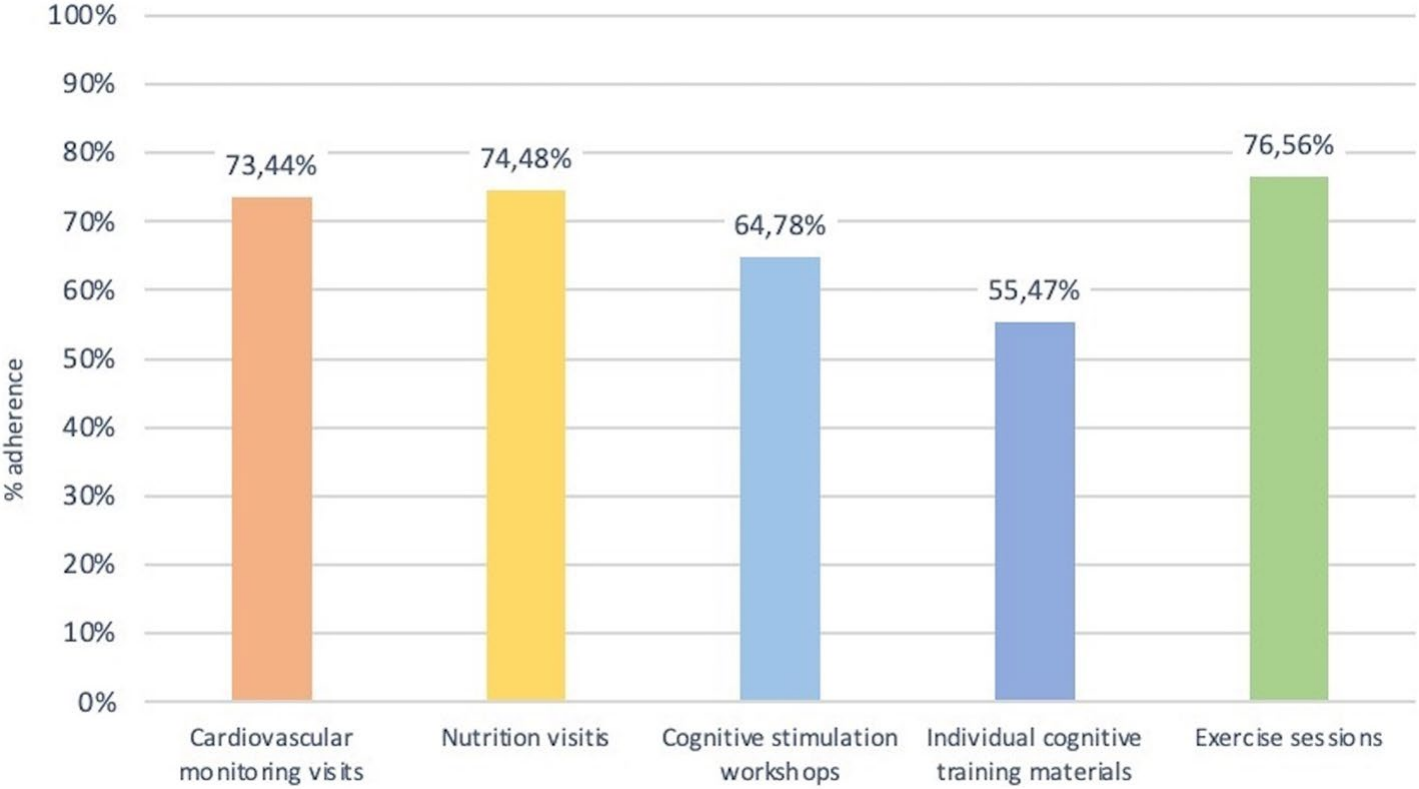
Mean adherence to each intervention component in the MD-Int group

Figure 3 shows the mean adherence to each intervention component for the participants in the MD-Int. group. The mean attendance to cardiovascular monitoring visits, nutritional workshops, and physical program activities was more than 70%. Adherence to cognitive intervention workshops and the completion of individual cognitive training materials were 64.8% and 55.5%, respectively.

Overall adherence to all intervention activities was at least “partial in 71.9% of the participants (Fig. 3). Thirty-five participants (54,7%) showed “high” adherence to the intervention plan. “Low” overall adherence was observed in 12 participants, mostly because of logistic and health issues. Owing to a lack of motivation, six participants did not adhere to any intervention activities.

At the final visit, the mean 12-month change for the NTB executive *z*-scores was significantly different between groups favoring the intervention (MD-Int group 0.11 (SD 0.43); RHA group − 0.13 (SD 0.48); *p* = 0.009) with a moderate Cohen’s *d* size effect of 0.52 (Table 3). At the post-intervention visit, the anxiety level was higher in the MD-Int group than in the RHA group, but still within the normal range (< cut-off of 11) (Table 3S). There was no difference in the cognitive performance between the groups in the mixed models of repeated measures (Tables 4S and 5S). However, 32 (64%) participants in the RHA and 22 (40%) in the MD-Int group declined in NTB executive function *z*-scores; 30 persons (61%) in the RHA and 22 (39%) in the MD-Int group declined in the NTB processing speed *z*-score (Table 4). The risk of decline was higher in the RHA group than that in the MD-Int group for the NTB executive function score (*p* = 019; odds ratio 2.57, 95% CI 1.13–5.84) and NTB processing speed score (*p* = .026, odds ratio 2.3, 95% CI 1.03–5.16) (Table 5).

**Table 3:** Mean change between the baseline and final visits

**NTBm *Z* scores**	**Regular health advice**		**Multidomain intervention**		***p***	***Effect size***
	*n*	*Mean (SD)*	*n*	*Mean (SD)*	***t-test***	***Cohen’s d***
**Global**	51	− 0.07 (0.33)	56	0.03 (0.32)	0.108	0.314
**Memory global**	50	0.02 (0.43)	56	0.01 (0.42)	0.899	0.025
**Executive Function**	50	− 0.13 (0.48)	55	0.11 (0.43)	0.009	0.520
**Processing speed**	49	− 0.08 (0.40)	55	− 0.01 (0.56)	0.451	0.149

*NTBm* Modified Neuropsychological Test Battery. Mean (SD) difference between *z* scores at pre-intervention and post-intervention visits in each group is shown

**Table 4:** Percentage of cognitive decline

NTBm scores	**Regular health advice**		**Multidomain intervention**		
		*Decline*		*Decline*	*p*
	*n*	*n* (%)	*n*	*n* (%)	*Fisher*
**Global**	51	25 (49)	56	26 (46)	0.848
**Memory global**	50	24 (48)	56	25 (45)	0.846
**Memory abbreviated**	47	25 (53)	56	26 (46)	0.555
Executive function	50	32 (64)	55	22 (40)	0.019
Processing speed	49	30 (61)	56	22 (39)	0.032

*NTBm* Modified Neuropsychological Test Battery. Decline = diff (*z* post − z pre) < 0; no decline = diff (*z* post − *z* pre) ≥ 0

**Table 5:** Risk of cognitive decline from pre-intervention to post-intervention

	**Odds ratio. Exp (B), CI: 95%**		***p*-value**
	**MD-Int (*n* = 56)**	**RHA (*n* = 51)**	
**Overall cognitive decline**			
NTBm total score	1 (reference)	1.065 (0.50–2.29)	0.871
**Cognitive decline per domain**			
NTBm memory global score	1 (reference)	1.145 (0.53–2.46)	0.729
NTBm memory abbreviated score	1 (reference)	1.311 (0.60–2.85)	0.494
NTBm executive functioning score	1 (reference)	2.583 (1.17–5.71)	0.019
NTBm processing speed score	1 (reference)	2.440 (1.11–5.36)	0.026

Cognitive decline was defined as a decrease in NTB scores between pre- and post-intervention assessments. Binary logistic analyses were carried out to assess the risk of cognitive decline in the regular health advice group compared with the multidomain intervention group

## Discussion

The GOIZ ZAINDU pilot trial has shown that a multidomain lifestyle and risk factor monitoring intervention to prevent cognitive decline in older adults at a high risk of dementia is feasible and reproducible. This experience has successfully demonstrated that the FINGER trial methodology is adaptable to Southern European conditions, including diet, exercise habits, and the health care system. Secondary efficacy analysis supports previous findings suggesting a protective effect of simultaneous intervention on cognition in different domains [12].

The feasibility concept is not as common in a clinical research context as in an economic or business management environment. We define feasibility in a clinical setting as the capacity to carry out the protocol and the degree of commitment by all the implicated institutions and participants. We argue that, with the GOIZ ZAINDU study, we have concluded that this multidomain lifestyle intervention can be adapted and implemented in our social, cultural, and institutional framework. As shown in Fig. 2, we observed a high degree of interest in any activity regarding cognitive decline and its prevention in older adults. In less than a month, almost 20% of the total population older than 60 years old in the Beasain municipality (*n*: 850) participated in the informative campaign performed by the local institutions. More than half of the participants in the informative sessions attended the screening invitation (*n*: 509). This is a meaningful result, considering data from multinational surveys indicating a low level of awareness among citizens regarding the possibility of ameliorating lifestyle and vascular health in order to prevent dementia [34].

The proportion of dropouts in the GOIZ ZAINDU pilot study was similar to that reported in previous studies [12–14]. Local adaptation of these types of multidomain intervention protocols may facilitate participant adherence [17, 35]. Adherence is also essential to ensure intervention program acceptance and efficacy. Furthermore, the overall adherence was better than that in previous multidomain intervention trials [36, 37]. Nevertheless, it is unclear which adherence is optimal for a lifestyle intervention. We tend to think that the more adherence, the more significant and better the effect on cognition. However, some data from the FINGER and MAPT study suggest most of the benefits observed in cognition was obtained by attending 50% of the intervention activities [38]. With the data from this pilot study, it is difficult to shed light on this issue; studies on a larger scale with more participants can help elucidate what degree of adherence is optimal in this type of intervention. In addition to adherence, the degree of compliance measured by changes in individuals’ lifestyles and correction of previous modifiable risk factors should be assessed. In the GOIZ ZAINDU study, owing to the small sample size, we could not measure it, but we propose to address this aspect within a larger efficacy study.

Together with the feasibility analysis, we must consider the implication of participating institutions in the economic sustainability of the project. For the GOIZ ZAINDU trial study, public and private resources were invested in this project, and rational usage played a crucial role in its implementation. Beasain town hall and Primary Health Care center involvement in the study activities ensure the sustainability of this health prevention initiative in the future. As an exploratory analysis, we observed a benefit in executive function, and processing speed was consistent with previously reported data from the FINGER trial. Nevertheless, these data should be considered cautiously, as the study design was not oriented to evaluate the efficacy of the intervention.

With this pilot experience, we have learned and drawn conclusions that beyond the efficacy and adherence data may help us design a clinical efficacy trial with a larger number of participants. On the one hand, the strategy of fluid and two-way communication with participants is essential for recruitment and promoting adherence to the study. On the other hand, the design of interventions tailored to the characteristics of the participants is also important, so that they are interesting for them and represent a fundamental change in their lifestyle. Finally, although it has been reported spontaneously, in our pilot study, many subjects also experienced a sensation of well-being by participating in group activities and increasing their previous degree of socialization. This is consistent with observational studies suggesting the benefits of healthy aging in promoting social engagement and psychological well-being. Observational studies have suggested the benefits of healthy aging, promoting social engagement, and psychological well-being [39]. Therefore, these conclusions should be considered in the design of future lifestyle interventions.

The GOIZ ZAINDU study results are consistent with previous data [12–14] and reinforce the importance of selecting an “at-risk-of-dementia” population for this type of interventions. These participants had a unique momentum in the cognitive decline continuum. These subjects were older adults with modifiable dementia risk factors who had already experienced slight changes in cognition. In the GZ study, we replicated the FINGER trial participant characteristics regarding cognitive variables by including brief cognitive testing in the screening period and then excluding participants with dementia symptoms [40]. It is never too early or too late in the lifespan to begin any initiative to promote cognition and healthy aging, especially in at-risk populations.

Beyond present and future drugs against pathophysiological targets of dementia, a holistic approach to dementia care is needed, especially for primary/secondary prevention. Sufficient evidence suggests that dementia should be addressed as a multifactorial entity [4], and a holistic approach is needed to promote healthy aging and dementia prevention (Geneva 2017) [41]. In 2019, the WHO published Guidelines for Cognitive Decline Risk Reduction based on a multicomponent approach. Although these guidelines are data-driven to date, they have not been consistently proven in appropriately designed trials. FINGER results hold the promise that healthy eating, exercise, and cognitive and social activities may have favorable effects on cognition, functional independence [42, 43], and health-related quality of life [34] and reduce the need for health care services [44] in older adults.

## Limitations

Lifestyle intervention trials have inherent limitations. Thus, previously healthier individuals usually adhere more to the prescribed activities [45]. Therefore, even in the RHA group, healthier people could follow the recommendations given better than the participants in the MD-Int group. with poorer adherence. Regarding this “healthy adherer” effect, we observed that the subgroup of MD-Int., the group of participants with high and partial adherence, had previous lower mean CAIDE dementia risk scores, higher mean MMSE scores, and lower proportion of MCI cases (Table 6S). Similarly, the control group also received recommendations on vascular risk factors, diet, and physical activity for ethical reasons. Therefore, this may have led to underestimation of the intervention effect.

## Conclusion

The GOID ZAINDU pilot study experience provides us with data to base an efficacy study, the CITA GO-ON trial (ClinicalTrials.gov Identifier: NCT04840030), which is currently part of the initiatives coordinated by WW-FINGER [46, 47], to deepen our knowledge of the mechanisms to prevent cognitive deterioration and promote healthy aging.

### Supplementary Information

**Additional file 1:** **Table 1S.** Adherence degrees to each intervention component. **Table 2S.** Baseline cognitive performance per group. **Table 3S.** Post-intervention characteristics per groups. **Table 4S.** Effect of intervention in cognitive change between pre-intervention and post-intervention visits per group. **Table 5S.** Effect of intervention in cognitive change between pre-intervention and post-intervention visits per groups. **Table 6S.** Baseline demographic, CAIDE, and Cognition characteristics differences between good and bad adherence groups. **Table 7S.** Cognitive domain z scores at pre-intervention and post-intervention visits. **Table 8S.** Cognitive z scores at pre-intervention and post-intervention visits per group. **Figure 1S.** Number of participants in each adherence category.

## Data Availability

The datasets used and/or analyzed during the current study are available from the corresponding author upon reasonable request.

## Abbreviations

FINGER: Finnish Geriatric Intervention Study to Prevent Cognitive Impairment and Disability
GZ: GOIZ ZAINDU
CAIDE: Cardiovascular Risk Factors, Aging and Dementia
MD-Int: Multidomain intervention
RHA: Regular health advice
MCI: Mild cognitive impairment
NTB: Neuropsychological Test Battery
NTBm: Modified Neuropsychological Test Battery
WHO: World Health Organization
MAPT: Multidomain Alzheimer Preventive Trial
PreDIVA: Prevention of Dementia by Intensive Vascular care
T@M: Memory alteration test
AD8: Alzheimer’s Disease 8 questionnaire
DSM-IV: Diagnostic and Statistical Manual of Mental Disorders, 4th edition
GP: General practitioners
MMSE: Mini-Mental State Examination
SD: Standard deviation
HAD: Hospital Anxiety and Depression Scale
CONSORT: Consolidated Standards of Reporting Trials
