# Supplementary material for: GOIZ ZAINDU study: a FINGER-like multidomain lifestyle intervention feasibility randomized trial to prevent dementia in Southern Europe
Source: Alzheimers Res Ther. 2024 Feb 27;16:44. doi: 10.1186/s13195-024-01393-z (PMC10898038; doi:10.1186/s13195-024-01393-z)
Supplement: Supplementary file 1 — Additional file 1: Table 1S. Adherence degrees to each intervention component. Table 2S. Baseline cognitive performance per group. Table 3S. Post-intervention characteristics per groups. Table 4S. Effect of intervention in cognitive change between pre-intervention and post-intervention visits per group. Table 5S. Effect of intervention in cognitive change between pre-intervention and post-intervention visits per groups. Table 6S. Baseline demographic, CAIDE, and Cognition characteristics differences between good and bad adherence groups. Table 7S. Cognitive domain z scores at pre-intervention and post-intervention visits. Table 8S. Cognitive z scores at pre-intervention and post-intervention visits per group. Figure 1S. Number of participants in each adherence category. [file 13195_2024_1393_MOESM1_ESM.docx]

**Supplementary material:**

| **Intervention** | **Level of adherence** | **number of participants** | **%** |
| --- | --- | --- | --- |
| Cardiovascular monitoring visits attendance | 3 visits | 37 | 57.81 % |
|  | 2 visits | 12 | 18.75 % |
|  | 1 visit | 6 | 9.38 % |
|  | 0 visits | 9 | 14.06 % |
| Nutrition visits attendance | 3 visits | 32 | 50.00% |
|  | 2 visits | 15 | 23.44 % |
|  | 1 visit | 17 | 26.56 % |
|  | 0 visits | 0 | 0 % |
| Cognitive stimulation workshops attendance | ≥70% | 37 | 57.80 % |
|  | ≥ 50 % | 45 | 70.30 % |
|  | ≥ 30 % | 49 | 76.60 % |
|  | < 30 % | 18 | 23.40 % |
|  | 0 workshops | 2 | 3.10 % |
| Individual cognitive training materials completed | ≥70% materials | 34 | 53.10 % |
|  | ≥ 50 % materials | 41 | 64.10 % |
|  | ≥ 30 % materials | 46 | 71.90 % |
|  | <30 % materials | 18 | 28.10 % |
|  | no material completed | 13 | 20.30 % |
| Physical exercise completed | ≥70% group visits | 25 | 39.10 % |
|  | individual exercise ≥ twice a week | 49 | 76.56 % |
|  | no exercise | 23 | 35.90 % |

Table 1S. Adherence degrees to each intervention component.

|  | Total Sample (*n=125)* | Regular Health Advice (*n* = 61) | Multidomain intervention *(n* = 64) | *p* |
| --- | --- | --- | --- | --- |
| **Characteristics at baseline** |  | | |  |
| **Memory** |  |  |  |  |
| WMS-III Logical Memory (immediate) | 9 (6 – 11) | 8.85 (4.09) | 8.42 (3.92) | .548 |
| WMS-III Logical Memory (delayed) | 7 (4 - 9) | 7 (4 - 10) | 6.50 (4 - 9) | .497 |
| CERAD Word List Learning | 15 (12 – 18) | 14.79 (3.85) | 15.02 (4.61) | .764 |
| CERAD Word List Recall | 4 (2 - 6) | 4 (2 - 5) | 4 (3 - 6) | .489 |
| WMS-R Visual Paired Associates (immediate) | 5 (3 - 8) | 6 (4 – 10) | 5 (3 - 8) | .103 |
| WMS-R Visual Paired Associates (delayed) | 4 (2 – 5) | 5 (2 - 6) | 3 (2 - 5) | .110 |
| **Executive Function** |  |  |  |  |
| CERAD Category Fluency | 14.78 (4.96) | 14.64 (5.38) | 14.91 (4.57) | .765 |
| WAIS-III Digit Span (total) | 11 (9 – 13) | 11 (9 - 13) | 11 (9 - 13) | .434 |
| CST (Condition C)* | 70 (43-100) | 71 (48 - 89) | 66 (30 - 101) | .822 |
| TMT shifting score (B-A)* | 143 (69 - 219) | 129 (70 - 211) | 160 (74.50 - 269) | .331 |
| Stroop test interference score (3-2)* | 37 (25- 48) | 37 (24 – 55.50) | 37 (25.25 – 58.75) | .749 |
| **Processing speed** |  |  |  |  |
| Letter Digit Substitution Test | 17 (10 - 21) | 16.38 (7.62) | 16.39 (7.03) | .998 |
| CST (condition A)* | 30 (25 - 41) | 28 (25.25 - 41) | 31.50 (24.25 - 41) | .713 |
| Stroop test (condition 2) | 37 (25 - 48) | 32 (27 - 39) | 33.50 (27.25 - 43) | .477 |
| MMSE | 26.50 (25.00 – 29.00) | 27 (25 - 29) | 26 (24 - 28) | .709 |

Table 2S. Baseline cognitive performance per group

Mean (SD) and median (Pc25-Pc75). Independent-samples t-test and Mann-Whitney test were applied. *Timed task where smaller number indicates faster performance/better test result. In other tasks bigger number indicates a better result. WMS-R: Wechsler Memory Scale-Revised; CERAD: Consortium to Establish a Registry for Alzheimer's Disease; CST: Concept Shifting Test; TMT: Trail Making Test; MMSE: Mini-Mental State Examination.

|  | Group | |  | |
| --- | --- | --- | --- | --- |
| Characteristics | Regular Health Advice  (*n* = 52) | Multidomain intervention *(n* = 56) | | *p* |
| Age | 75.25 (11.81) | 75.93 (6.42) | | .709 |
| Sex: women | 31 (60%) | 35 (63%) | | .844 |
| Education: years | 7.81 (2.92) | 8.57 (3.99) | | .262 |
| Anxiety (HADS) | **4.98 (3.17)** | **6.54 (4.13)** | | **.033** |
| Depression (HADS) | 3.73 (3.57) | 4.45 (3.67) | | .307 |

HADS: Hospital Anxiety and Depression Scale

Table 3S. Post-intervention characteristics per groups

|  | **Z Global score** | | **Z Memory score** | | **Z Executive Functioning score** | | **Z Processing Speed score** | |
| --- | --- | --- | --- | --- | --- | --- | --- | --- |
|  | **Fixed Coeff.** | **p-value** | **Fixed Coeff.** | **p-value** | **Fixed Coeff.** | **p-value** | **Fixed Coeff.** | **p-value** |
| Group = 1 | -0.060 | 0.685 | -0.070 | 0.675 | -0.164 | 0.322 | 0.030 | 0.893 |
| Visit = 2 | -0.032 | 0.868 | 0.135 | 0.538 | -0.217 | 0.307 | 0.034 | 0.897 |
| Group = 1 * Visit = 2 | 0.143 | 0.584 | -0.027 | 0.926 | 0.413 | 0.144 | 0.039 | 0.911 |
| Constante | -0.354 | 0.002 | -0.372 | 0.003 | -0.207 | 0.097 | -0.443 | 0.009 |
| AIC corrected |  | 251.678 |  | 272.205 |  | 265.352 |  | 313.621 |

*1 = Multidomain Intervention, 0 = Regular Health Advice (reference); Visit 2 = post-intervention assessment, Visit 1 = pre-intervention assessment (reference)*

Table 4S. Effect of intervention in cognitive change between pre-intervention and post-intervention visits per group.

|  | **Z Global score** | | **Z Memory score** | | **Z Executive Functioning score** | | **Z Processing Speed score** | |
| --- | --- | --- | --- | --- | --- | --- | --- | --- |
|  | **Fixed Coeff.** | **p-value** | **Fixed Coeff.** | **p-value** | **Fixed Coeff.** | **p-value** | **Fixed Coeff.** | **p-value** |
| Anxiety | 0 | <0.001 | 0.019 | 0.178 | 0 | <0.001 | -0.020 | 0.181 |
| Education | 0.09 | <0.001 | 0.082 | <0.001 | 0.091 | <0.001 | 0.117 | <0.001 |
| Group = 1 | -0.15 | 0.195 | -0.155 | 0.269 | -0.206 | 0.08 | -0.139 | 0.356 |
| Visit = 2 | 0.004 | 0.975 | 0.048 | 0.752 | -0.078 | 0.54 | 0.067 | 0.665 |
| Group= 1 * Visit = 2 | 0.125 | 0.476 | 0.067 | 0.752 | 0.238 | 0.177 | 0.044 | 0.839 |
| Constante | -0.691 | <0.001 | -0.702 | <0.001 | -0.682 | <0.001 | -0.808 | <0.001 |
| AIC corrected |  | 487.248 |  | 556.173 |  | 477.284 |  | 558.351 |

*Anxiety and Education as continuous variables. 1 = Multidomain Intervention, 0 = Regular Health Advice (reference); Visit 2 = post-intervention assessment, Visit 1 = pre-intervention assessment (reference).*

Table 5S. Effect of intervention in cognitive change between pre-intervention and post-intervention visits per groups.

|  | **Overall adherence** | |  |  |
| --- | --- | --- | --- | --- |
|  | **High and partial adherence participants (n: 46)** | **Low and very low adherence participants (n: 18)** |  | *p* |
| **Demographic characteristis** |  |  |  |  |
| Age | 74.90 (6.57) | 76.06 (5.48) |  | 0.433 |
| Sex: woman, n (%) | 27 (58.70%) | 10 (55.60%) |  | 0.819 |
| Education (years) | 8.85 (3.94) | 7.56 (4.49) |  |  |
| Occupation |  |  |  |  |
| Upper | 8 (0.17) | 2 (0.12) |  | 0.854 |
| Middle | 16 (0.35) | 6 (0.35) |  |  |
| Lower | 22 (0.48) | 9 (0.53) |  |  |
| **CAIDE dementia risk score** | 8.3 (1.93) | 9.33 (2.03) |  | 0.05 |
| **Cognition** |  |  |  |  |
| Mild Cognitive Impairment (%) | 13 (28.30 %) | 12 (66.70 %) |  | 0.005 |
| Mini Mental State Examination | 26.71 (2.64) | 24.61 (3.11) |  | 0.015 |

Table 6S. Baseline demographic, CAIDE, and Cognition characteristics differences between good and bad adherence groups.

**NOT INCLUDED IN THE TEXT:**

|  | PRE-INTERVENTION | POST-INTERVENTION |
| --- | --- | --- |
| NTBm scores | ALL (*n* = 125)  mean (SD) | ALL (n = 107)  Mean (SD) |
| **Global** | -0.03 (0.69) | 0.02 (0.78) |
| **Memory global** | -0.00 (0.79) | 0.09 (0.87) |
| **Memory abbreviated** | -0.01 (0.82) | 0.13 (0.89) |
| **Executive Function** | -0.04 (0.69) | -0.01 (0.77) |
| **Processing speed** | -0.01 (0.88) | 0.07 (0.89) |

NTBm: Neuropsychological Test Battery modified.

Table 7S. Cognitive domain z scores at pre-intervention and post-intervention visits.

|  | Regular Health Advice | |  | Multidomain Intervention | |
| --- | --- | --- | --- | --- | --- |
| Cognitive domain | Pre | Post |  | Pre | Post |
| (m: z score) | n = 61 | n = 51 |  | n = 64 | n = 56 |
| **Global** | 0.02 (0.67) | -0.03 (0.81) |  | -0.07 (0.72) | 0.06 (0.76) |
| **Memory global** | 0.04 (0.74) | 0.09 (0.86) |  | -0.04 (0.84) | 0.09 (0.89) |
| **Memory abbreviated** | 0.11 (0.77) | 0.16 (0.88) |  | -0.09 (0.87) | 0.10 (0.89) |
| **Executive Function** | 0.04 (0.67) | -0.10 (0.86) |  | -0.10 (0.72) | 0.07 (0.68) |
| **Processing speed** | 0.03 (0.84) | 0.07 (0.81) |  | -0.05 (0.92) | 0.06 (0.98) |

Mean (SD). NTBm: Neuropsychological Test Battery modified. Z scores standardized to the baseline mean and SD. Pre: pre-intervention assessment, Post: post-intervention assessment.

Table 8S. Cognitive z scores at pre-intervention and post-intervention visits per group.

Figure 1S. Number of participants in each adherence category
